# Computational models for contact current dosimetry at frequencies below 1 MHz

**DOI:** 10.1007/s11517-020-02284-9

**Published:** 2020-12-02

**Authors:** Pia Schneeweiss, Dorin Panescu, Dominik Stunder, Mark W. Kroll, Christopher J. Andrews, Tobias Theiler

**Affiliations:** 1 Research Center for Bioelectromagnetic Interaction (femu), Institute for Occupational, Social and Environmental Medicine, Uniklinik RWTH Aachen University, Aachen, Germany; 2 Advanced Cardiac Therapeutics, Inc., Santa Clara, CA USA; 3 Department of Biomedical Engineering, University of Minnesota Twin Cities, Minneapolis, MN USA; 4 University of Queensland, Brisbane, Australia

**Keywords:** Computer simulations, Accidental electrocution, Dosimetry, Contact currents, Impedance

## Abstract

Electric contact currents (CC) can cause muscle contractions, burns, or ventricular fibrillation which may result in life-threatening situations. In vivo studies with CC are rare due to potentially hazardous effects for participants. Cadaver studies are limited to the range of tissue’s electrical properties and the utilized probes’ size, relative position, and sensitivity. Thus, the general safety standards for protection against CC depend on a limited scientific basis. The aim of this study was therefore to develop an extendable and adaptable validated numerical body model for computational CC dosimetry for frequencies between DC and 1 MHz. Applying the developed model for calculations of the IEC heart current factors (HCF) revealed that in the case of transversal CCs, HCFs are frequency dependent, while for longitudinal CCs, the HCFs seem to be unaffected by frequency. HCFs for current paths from chest or back to hand appear to be underestimated by the International Electrotechnical Commission (IEC 60479-1). Unlike the HCFs provided in IEC 60479-1 for longitudinal current paths, our work predicts the HCFs equal 1.0, possibly due to a previously unappreciated current flow through the blood vessels. However, our results must be investigated by further research in order to make a definitive statement. Contact currents of frequencies from DC up to 100 kHz were conducted through the numerical body model Duke by seven contact electrodes on longitudinal and transversal paths. The resulting induced electric field and current enable the evaluation of the body impedance and the heart current factors for each frequency and current path.

## Introduction

Electric contact currents (CCs) occur when one or more body parts (e.g., the hand) touch a conducting object with an electric potential that is different from that of the body. Protection against CCs at frequencies below 1 MHz is needed to prevent painful muscle contractions, burns, or ventricular fibrillation (VF), which is the main cause of deaths by CCs [1]. Additionally, secondary injuries and psychological problems can be triggered [2–7]. Since the myocardial cells become more and more insensitive with increasing frequencies, the tissue is more likely to be burned before it is excited at higher frequencies [8].

The risk assessment of CC dates from the mid-1900s. Osypka [9] and Dalziel and Lee [10–12] contributed by determining the total body current thresholds marking the perception, let-go, and VF thresholds. The threshold of perception marks the minimal value of the current through the body that can be perceived by a person. The threshold of let-go describes the maximal value of the current through the body at which an individual is able to release the energized contact area. There is no let-go threshold for direct currents, only at the start and the end of the current flow leads to painful and seizure-like muscle contractions. In the early 1970s, the International Electrical Commission (IEC) combined these physiological thresholds with body impedance measurements from Freiberger [13] to derive a set of international standards [1, 14–16]. Since then, these standards have been updated several times based on results from studies such as those of Biegelmeier et al. [17, 18]. Since introducing the so-called heart current factor (HCF) [19], these standards consider the dependence of the VF risk on current paths, taking into account that, e.g., a current path from hand to feet drives a larger portion of the total body current through the heart (higher HCF) than a current path from foot to foot (lower HCF). In other words, VF is more likely to occur in a person when the current enters the body through the hand and exits through the feet than when the current enters the body through one foot and exits through the other foot. IEC 60479-1 gives HCFs to scale risks of VF for 16 different current paths [1]. Furthermore, the International Commission on Non-Ionizing Radiation Protection [20] and the Institute of Electrical and Electronics Engineers (IEEE) address the effect of CCs in their recommendations [20–22].

The scientific basis for standards on CCs is very limited, especially regarding frequencies other than DC and 50/60 Hz as well as in the number of study participants [1]. In vivo experiments with CC are rare due to potentially hazardous or painful effects for participants. Important internal field distribution measurements of vulnerable body areas were only carried out using body phantoms [9] and in cadaver studies [13, 23], where probes were inserted into the body around the heart in order to estimate HCFs. The outcome is therefore limited to cadaver tissue’s electrical properties as well as to the size, position, and sensitivity of the utilized probes.

Due to these limitations in experimental studies, numerical approaches were proposed and carried out. Early studies of Brucher et al. [24–26] and Biegelmeier et al. [27] lacked investigations of frequencies other than 50/60 Hz, high-resolution body models, and tissue segmentations. Studies using high-resolution anatomical body models, scrutinize intermediate and radio frequency currents on current paths from fingertip to feet [28, 29], validate their numerical methods with analytical models [29, 30], and assess skeletal muscle anisotropies and skin properties at 50 Hz [30]. These studies, however, show neither body model validation attempts with in vivo data nor evaluations of different current paths as suggested by the IEC [1]. Only two studies [31, 32] considered different current paths (HCFs), but both were limited to 50 Hz. Additionally, they lack validation of the used body models and numerical method. In general, there exists no peer-reviewed literature on HCFs besides 50 Hz studies, even though VF can occur in a wide frequency range (DC to 100 kHz) [8].

To address this limitation, our study presents computational methods for dosimetric studies on CCs using a resistive-capacitive high-resolution anatomical body model in the frequency range from DC to 1 MHz focusing on internal field distributions and the potential risk of VF. Based on a recent research agenda published by Reilly, Hirata [33], in this study, special attention was directed to model validation, tissue anisotropy, and skin properties. The aim was to develop an extendable and adaptable validated toolbox for computational dosimetry studies on CCs enabling assessments based on the generated data sets using electrical cell-stimulation modeling and future investigation with new body models.

## Methods

### Simulation setup

The computational simulation platform Sim4Life version 2.2.3 and the high-resolution anatomic body model “Duke” [34] version 3.1 were used for all simulations applying Sim4Life’s “Electro Quasi-Static” solver based on the finite element method. The Electro Quasi-Static solver is applicable for the calculation of electric fields in the (extremely) low-frequency range (*f* < 1 MHz) and considers both the conductivity and the relative permittivity of the body tissues.

Sim4Life was running on a workstation with 2 Intel Xenon CPUs E5-2650v3 (2.3 GHz) and 128 GB RAM. The voxel edge length of Duke was set to 1 mm in the heart, and 2–3 mm for the remaining tissue. The maximum edge length relation relaxation gives the percentage of local relaxation of the grading ratio to increase the dynamics of the gridder without risking over-refinement in areas with closely spaced grid lines. It was set to 20% to ensure a highly homogenous resolution in the thorax region.

Overall, seven contact electrodes (100 × 100 mm) were positioned according to the IEC [1, 23] positions at the right/left hand (RH/LH), right/left foot (RF/LF), posterior thorax (PT), anterior thorax (AT), and (posterior) seat [35]. All 16 IEC current paths can be simulated with the seven contact electrodes. The current paths are abbreviated in the following manner:

- A current path from the RH to the LH is abbreviated as RH-LH.
- A current path from both hands (BH) to both feet (BF) is abbreviated as BH-BF.
- A current path from LH to any combination of foot or feet is abbreviated as LH-xF.

The CC scenario was implemented as a voltage-driven problem, meaning the voltage level (touch voltage) of the touched conductive object (here the contact electrodes) was set to a fixed value/amplitude and the resulting total body current (*I*_*Body*_) was measured with four surface integrals over the numerically derived internal electric field. Through Ohm’s law, the total body impedance (*Z*_*Total*_) is quantifiable for every current path, touch voltage, and current frequency.

The validity of body model’s electrical properties is described in the following subsections. If not stated otherwise, the body models’ segmented tissues were linked to the tissue electrical properties database version 2.7 of the Foundation for Research on Information Technologies in Society (IT’IS) [36], which mainly refers to Gabriel et al. [37–40]. This database provides conductivity and relative permittivity values for all used tissues as a function of frequency.

### Skeletal muscle anisotropy

The electrical properties of human skeletal muscles are anisotropic at low frequencies (*f* < 1 MHz), meaning they differ considerably with the direction of the current [35, 38]. The conductivity of skeletal muscle is therefore often measured along (longitudinal) and across (transversal) the muscle fibers. This definition of longitudinal and transversal must not be confused with the longitudinal or transversal current paths through the body. Since the skeletal muscles and therefore also the muscle fibers are oriented proximally or distally (e.g., wrist root to elbow for the forearm or ankle to knee for the lower leg), the main part of the CCs is expected to traverse the skeletal muscles longitudinally. Prior numeric assessments [30, 41, 42] of skeletal muscle assumed anisotropy ratios (longitudinal/transversal) of between 1.7 and 2 for 50 Hz currents, which lies within the range of literature values reviewed by Gabriel et al. [38]. Thus, they show no major differences between an anisotropic setup and an isotropic setup using the longitudinal conductivity value [30]. Thus, the skeletal muscle fiber tissue was set to the longitudinal conductivity values according to Gabriel et al. [37].

### Body models’ internal and total impedance

The total body impedance *Z*_*Total*_ of humans can be split into the skin impedance (*Z*_*Skin*_) of the surface area which is in contact with the voltage source (i.e., contact area) in series with the internal body impedance *Z*_*Intern*_. The internal body impedance is mostly dependent on the current path, and is mainly resistive and its change with frequency is negligible [1]. *Z*_*Skin*_ is often described as a resistive-capacitive network and its impedance decreases with rising frequencies (*f*). This model has been used and proven correctly in numerous studies and was therefore also implemented in the safety standards IEC 60479-1[1] and IEC 60990 [43] with defined values for the different elements of the RC circuit. *Z*_*Skin*_ drops significantly above 10 kHz. In cases of CCs with extremely low frequencies (ELF; *f* < 1 kHz) the skin impedance is strongly dependent on the touch voltage *U*_*t*_. At higher voltages (*U*_*t*_ > 220 V) the skin impedance decreases drastically and becomes negligible when the current forces skin breakdown [1, 17]. Thus, at frequencies higher than 10 kHz and touch voltages higher than 220 V, *Z*_*Total*_ approximates *Z*_*Intern*_. At frequencies between 1 and 10 kHz, the question of whether or not the skin impedance is negligible needs to be assessed on a case by case basis.

In view of *Z*_*Total*_ as the limiting factor for the total body current (*I*_*Body*_), the dependency of *Z*_*Skin*_ on the touch voltage and the frequency becomes a key factor in the modeling of CCs [44]. In order to cover exposure scenarios where *Z*_*Skin*_ needs to be considered and where it can be neglected, two body models were developed. The Internal Body Model (Intern-BM) considers only *Z*_*Intern*_ and the Total Body Model (Total-BM) considerers both *Z*_*Intern*_ and *Z*_*Skin*_. An overview of the body model classification for different accident scenarios is given in Table 1.

**Table 1:** Body model classification for different exposure scenarios

	DC ≤ *f* ≤ 1 kHz (ELF)	1 kHz ≤ *f* ≤ 10 kHz	*f* > 10 kHz
25 V ≤ *U*_*t*_ ≤ 220 V (skin intact)	Total-BM	Indiv. cases	Intern-BM
*U*_*t*_ *>* 220 V (skin break down)	Intern-BM	Intern-BM	Intern-BM

### Total-BM and intern-BM implementation

For the Total-BM, the electrical properties of the skin were adapted to match the impedance values derived from IEC 60479-1 [1]. In order to achieve that, the skin at the contact areas was removed and the touch voltage and frequency-dependent electric properties of the skin were implemented as a 2-mm thick slice of the contact electrode resembling *Z*_*Skin*_ (cf. Table 2). In that way, it is possible to control the electrical properties of *Z*_*Skin*_ at every position of the electrode and to ensure an exact contact of 10 × 10 cm^2^. Following IEC 60479-1, this is defined as a “large surface area.” The remaining material of the contact electrode was assigned to a value of 1.0 Sm^−1^ in order to be magnitudes higher than the skin conductivity value at ELF of 0.2 mSm^−1^ [36]. At 1.0 Sm^−1^, the influence of the remaining material can be considered insignificant for the assessed parameters (*Z*_*Intern*_, *Z*_*Total*_, and the HCF). A higher conductivity value causes solver instability because it spreads the range of material parameters beyond the greatest possible dynamic range of the solver. The results for voltage and frequency-dependent skin conductivity are listed in Table 2.

**Table 2:** Electric properties of *Z*_*Skin*_ for different touch voltage amplitudes

	25 V	50 V	100 V	220 V
Conductivity (mS m^−1^)	0.14	0.19	0.27	0.85
Relative permittivity	2.4e4	3.9e4	6.4e4	13.2e4

For the Intern-BM, the 2-mm layer resembling *Z*_*Skin*_ was removed so that the touch voltage is in direct contact with tissue underlying the skin, enabling simulations of exposure scenarios where the skin impedance influence is considered negligible (cf. Table 1). The contact area varies with the contact electrode positions between 70 and 100 cm^2^ due to the three-dimensional body surface. However, *Z*_*Intern*_ is not dependent on the contact area and therefore the contact area can vary for the Intern-BM [1].

**Fig. 1:**
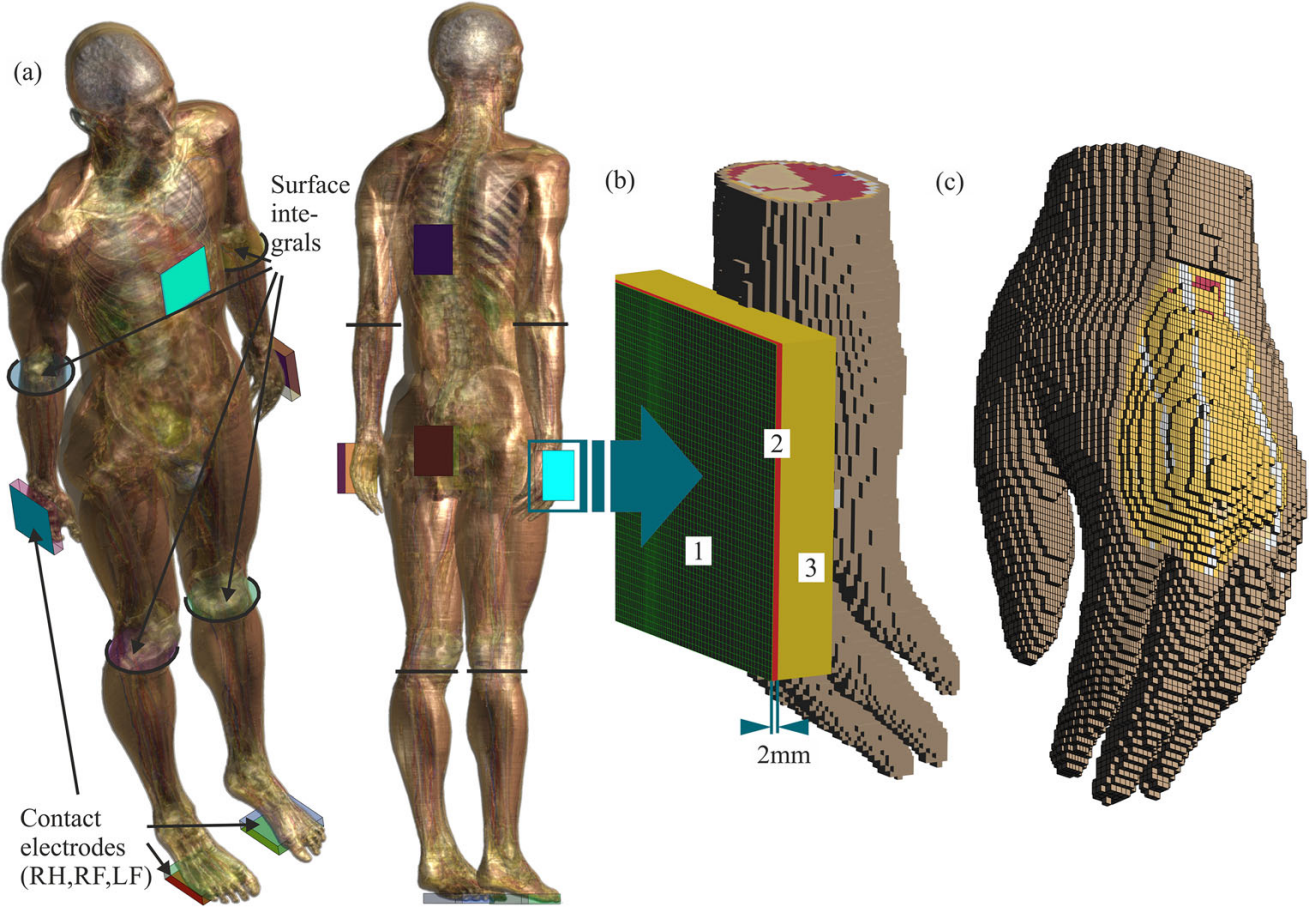
**a** Duke anatomic body model with the seven contact electrodes and the four surface integrals for the total body current (*I*_*Body*_) measurement. **b** Voxels view of the LH with its contact electrode and the simulation setup. 1. Boundary condition (*U*_*t*_ on green grid), 2. Red slice resembles *Z*_*Skin*_, which is removed when using Intern-BM 3. Contact electrode material with 1 Sm^−1^. **c** Voxels of the LH and the contact area where the skin is removed and the underlying tissue (e.g., subcutaneous adipose tissue; yellow) is visible

Figure 1 gives an overview of all model features. Of note, for studies focusing on tissues directly underlying the skin, this method is most likely unsuitable.

### Model validation

In order to validate the presented approach, the net electrical properties of the body models (the total body impedance *Z*_*Total*_) were calculated and compared to in vivo body impedance measurement values from literature either considering different touch voltages [1, 13, 17] or higher frequencies [47, 48]. All these studies used similar exposure conditions (e.g., current path: RH-LH and RH-BF, contact area) enabling the comparison with the Intern- and the Total-BM.

### HCF simulations

For every current path, a different portion of the total current is being driven through the heart. To consider this effect HCFs were introduced [1]. HCFs are therefore important for determining the risk of VF due to CCs in different paths. The measurements of Sam [19] are of special importance in this context since they are the literature basis for the IEC [1] HCF values. Sam [19] applied CCs due to 220 V touch voltages to human cadavers. He compared the influence of DC and 50 Hz AC for different voltages on the body resistance and concluded that for voltages above 100 V, the difference is negligible. Therefore, he inserted six electrodes at the base, apex, front, back, top, and bottom of the cadavers’ hearts and measured three orthogonal voltages across the heart (base to apex, front to back, and top to bottom) at 50 Hz. By dividing the measured voltages by the distance of the electrodes (85–160 mm), Sam approximated the electric field (EF) vector (*E*_*Sam*_) for the heart region by assuming a unidirectional homogenous EF distribution and a negligible error due to differing electrode distances. A heterogeneous EF distribution is not assessable with Sam’s method because of the averaging effect due to the probe size and locally changing EF vector orientations.

Sam calculated his HCF (*F*_*Sam*_) for a given current path *p* by dividing *E*_*Sam*_ by the total body current (*I*_*Bod*_) to eliminate the effects of changing body impedances due to the different current paths. For simplicity, this term is here normalized on the current path LH-BF (*p*_*LH–BF*_) at a given frequency, because Sam did not consider a frequency dependency in his equation, as explained in the previous paragraph.

1 $$ {F}_{Sam}(p)=\frac{E_{Sam}(p){I}_{Body}\left({p}_{LH- BF}\right)}{I_{Body}(p){E}_{Sam}\left({p}_{LH- BF}\right)} $$

**Fig. 2:**
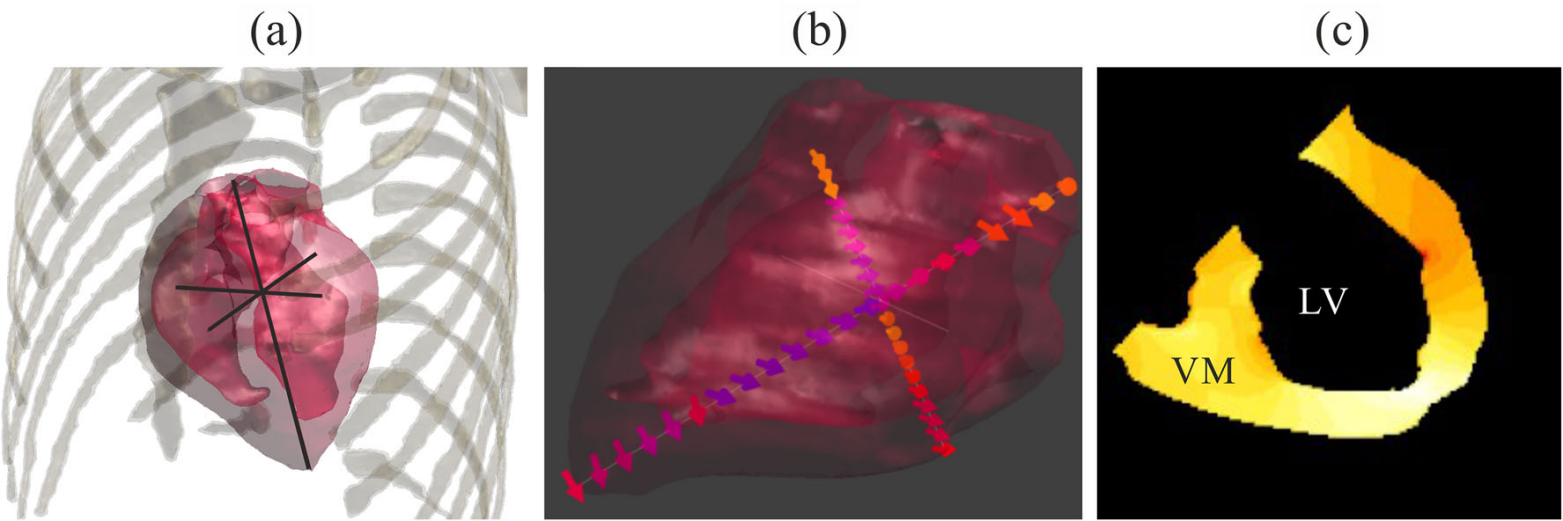
**a** Computational imitation of Sam [19]. Thorax bones, heart muscle, and three orthogonal line integrals as imitation of Sam’s voltage measurements. **b** The electric field vectors along the line integral from base to apex and top to bottom. **c** Cross-section slice parallel to the frontal plane

Sam’s [19] HCF measurements were modeled numerically in order to compare the results of our numerical model (cf. Fig. 2) with the cadaver measurements. Three orthogonal line integrals modeling the voltage measurements were implemented into the computational model with a local resolution of 1 mm (cf. black lines in Fig. 2(a)). For clarification, Fig. 2(b) shows the electric field vectors along two of the three line integrals. The HCF of the numerically modeled Sam method will be referred to with *F*_*SamNum*_.

The voxels of the ventricular myocardium (VM) and the left ventricle lumen (LV) are shown. The EF of the ventricle for every voxel in the slice is displayed. It is shown how only the ventricle myocardium voxels of this slice are selected (highlighted) as an example of how the data for *E*_99*Heart*_ is selected for all the slices of the heart.

For our study, a novel HCF assessment method was developed taking advantage of numerical simulations being able to provide the local EF distribution in the heart muscle. Therefore, the 99th percentile (cf. guidelines from the International Commission on Non-Ionizing Radiation Protection (ICNIRP [20])) of the EF distribution in the ventricle myocardium (*E*_99*Heart*_) was calculated for all 16 current paths (cf. Section 2.1) and five different frequencies (DC, 50 Hz, 1 kHz, 10 kHz, 100 kHz). Accordingly, *E*_99*Heart*_ (*p, f*) is noted as a function of current path and frequency. To enable a comparison to Sam’s [19], the numerical HCF (*F*_99*Num*_) is also normalized on the current path LH-BF (*p*_*LH–BF*_) (cf. (1) and (2)).

2 $$ {F}_{99\kern0.1em Num}\left(p,f\right)=\frac{E_{99\kern0.1em Heart}\left(p,f\right)}{I_{Body}\left(p,f\right)}\frac{I_{Body}\left({p}_{LH- BF},f\right)}{E_{99\kern0.1em Heart}\left({p}_{LH- BF},f\right)} $$

In order to clarify which part of the heart muscle was considered, the corresponding electric field of a slice of the ventricle myocardium is shown in Fig. 2(c). The slice is orientated parallel to the frontal plane. For *E*_99*Heart*_, the whole ventricle myocardium was considered, since this is the part of the heart muscle where fibrillation is induced.

All HCF methods were applied on all 16 current paths defined in Section 2.1. The Intern-BM was used for all HCF calculations, because the skin impedance has no influence on the HCF.

## Results and discussion

### Model validation

**Fig. 3:**
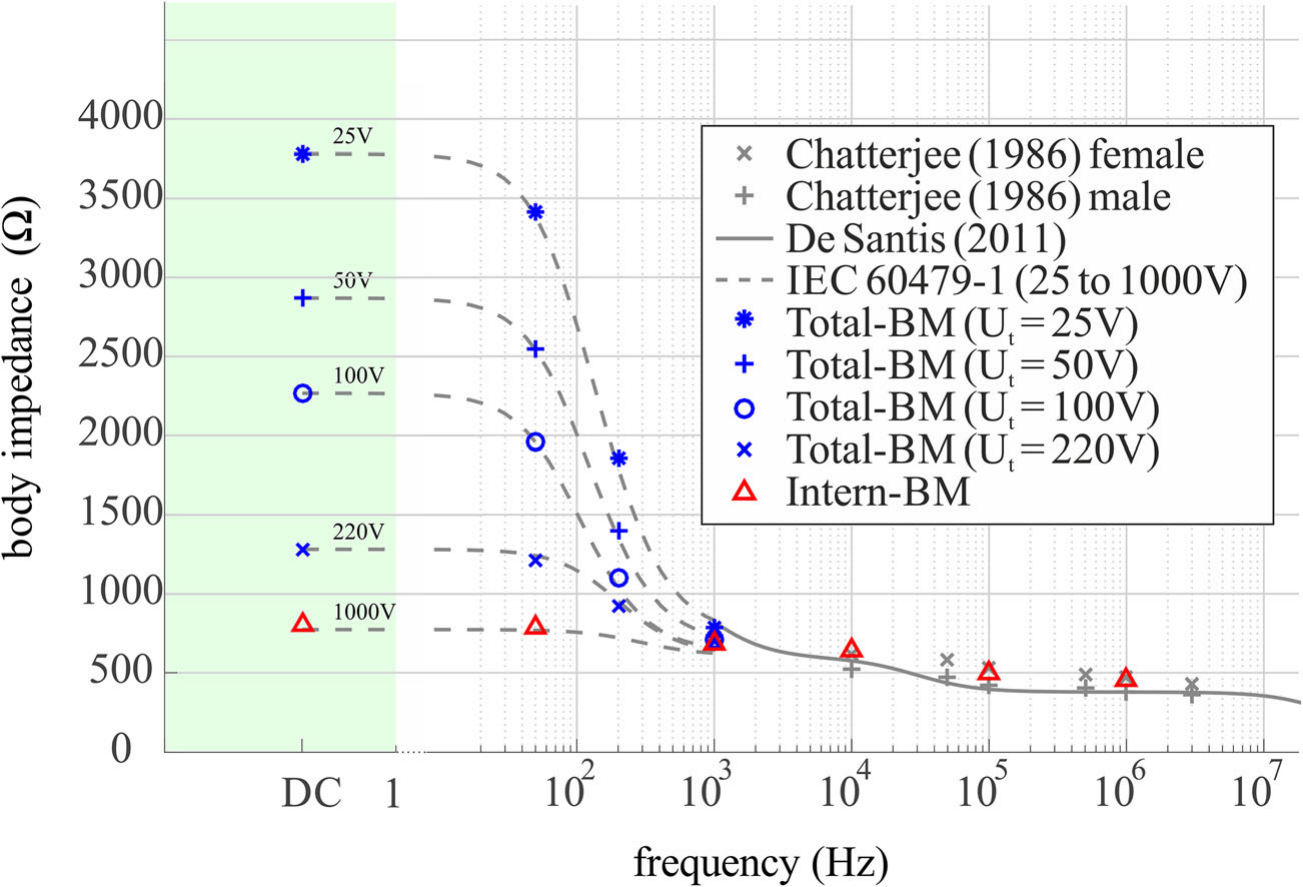
Comparison of the net impedance of the body models with literature impedance values for validation purposes. Compliance is achieved for both developed models

Figure 3 demonstrates the validation of the net impedance of the body models developed in this study. The Intern-BM (red triangles) is consistent with the literature values where the skin’s impedance contribution to *Z*_*Total*_ is negligible, either due to high touch voltages (cf. IEC 1000 V curve) or frequencies above 1 kHz (cf. De Santis or Chatterjee curves). The exceeding of De Santis values by the Intern-BM originates from the natural variations of biological tissues due to physiological processes or other functional requirements. De Santis [48] himself mentions a large variability of his results in his conclusion, however, lacks providing the standard deviation. The spread of the dielectric values from Gabriel et al. [45] used for the BM presented in this paper ranges from about ± 5–10% above 100 MHz to ± 15–25% at the lower end of the frequency scale [39]. Thus, our calculated values are within the statistically explainable deviation. The Intern-BM’s net impedance is therefore validated by the impedance measurements provided in the current literature.

The IT’IS tissue database does not consider the voltage dependency of the skin impedance at the contact area. Therefore, based upon the validated Intern-BM, the electrical properties of *Z*_*Skin*_ needed to be found for the Total-BM. The deeply founded model of an RC circuit as *Z*_*Skin*_, as described in Section 2.3, was used to vary the electrical properties of its different elements for the best fitting model considering frequencies up to 1 kHz. The electrical properties of *Z*_*Skin*_ shown in Table 2 led to compliance (cf. Total-BM in Fig. 3) with the impedance values derived from the IEC [1] for every chosen frequency between DC and 1 kHz. The results also comply with the general understanding on the capacitive properties of the skin for low voltages [46, 49]. The influence of the touch voltage is very large. The conductivity increases by a factor of 6 between 25 and 220 V touch voltage.

In general, the comparison of the total body impedance with literature values (cf. Fig. 3) confirms the validity of the net electrical tissue properties Intern-BM. The IEC [1] values additionally enable the validation of the voltage and frequency-dependent Total-BM, providing in return insights into the conductivity and relative permittivity of the skin under different exposure situations (cf. Table 2). Beside the skin, the electrical properties of other specific tissues are not directly assessable, but the agreement of the body models’ total body impedance with the literature is a clear indication that the individual tissue properties are also valid.

### Numerical HCF of longitudinal current paths

Table 3 shows the numerical HCFs (*F*_*SamNum*_ and *F*_99*Num*_ as defined in method Section 2.6) determined in this study for xH-xF and PS-xH current paths. In this table, only the current paths which create longitudinal currents (current flowing lengthwise) in the trunk of the human body are shown. For comparison, *F*_*Sam*_ [19] HCF values that are the currently valid values provided in IEC 60479-1 [1] are shown in the second column. The HCFs are given from DC to 100 kHz as this is the spectrum for inducing VF. As defined in Section 2.6, the first row equals 1.0 because *F*_99*Num*_ is normalized to the current path LH-BF for each frequency.

**Table 3:** *F*_99*Num*_ according to (2) for current paths generating longitudinal currents

Path	*F*_*Sam*_ at 50 Hz	*F*_*SamNum*_	*F*_99*Num*_				
		50 Hz	50 Hz	1 kHz	10 kHz	100 kHz	DC
LH-BF	1.00	1.00	1.00	1.00	1.00	1.00	1.00
LH-LF	1.00	1.00	1.01	1.01	1.01	1.01	1.01
LH-RF	1.00	1.00	0.99	0.99	0.99	0.99	0.99
BH-BF	1.00	0.96	1.00	1.00	0.99	0.96	1.02
RH-BF	0.80	1.02	1.02	1.02	1.00	0.95	1.06
RH-LF	0.80	1.03	1.03	1.03	1.01	0.97	1.07
RH-RF	0.80	1.01	1.01	1.00	0.98	0.94	1.04
PS-LH	0.70	1.00	1.01	1.00	1.00	1.00	1.01
PS-RH	0.70	1.02	1.02	1.02	1.00	0.95	1.06
PS-BH	0.70	0.96	1.01	1.00	0.99	0.96	1.03

For the current paths shown in Table 3, the deviations of *F*_*SamNum*_ and *F*_99*Num*_ from 1.0 are negligible. They are also in accordance with the *F*_*Sam*_ values for the current paths LH-xF and BH-BF. For RH-xF and PS-xH current paths, however, *F*_99*Num*_ and *F*_*SamNum*_ deviate from the literature values [1].

The compliance of *F*_*SamNum*_ with *F*_99*Num*_ shows that the error due to the probe size in Sam’s method (described in Section 2.6) is not important for longitudinal current paths. Thus, for these current paths, the differences between Sam’s measurements and our numerical findings must be due to the differences of our numerical body model and the cadaver Sam used. A discussion about the quality of both approaches is necessary, because the IEC [1] results, which origin from Sam [19], have been accepted and applied in practice for decades. They should therefore not be dismissed easily.

**Fig. 4:**
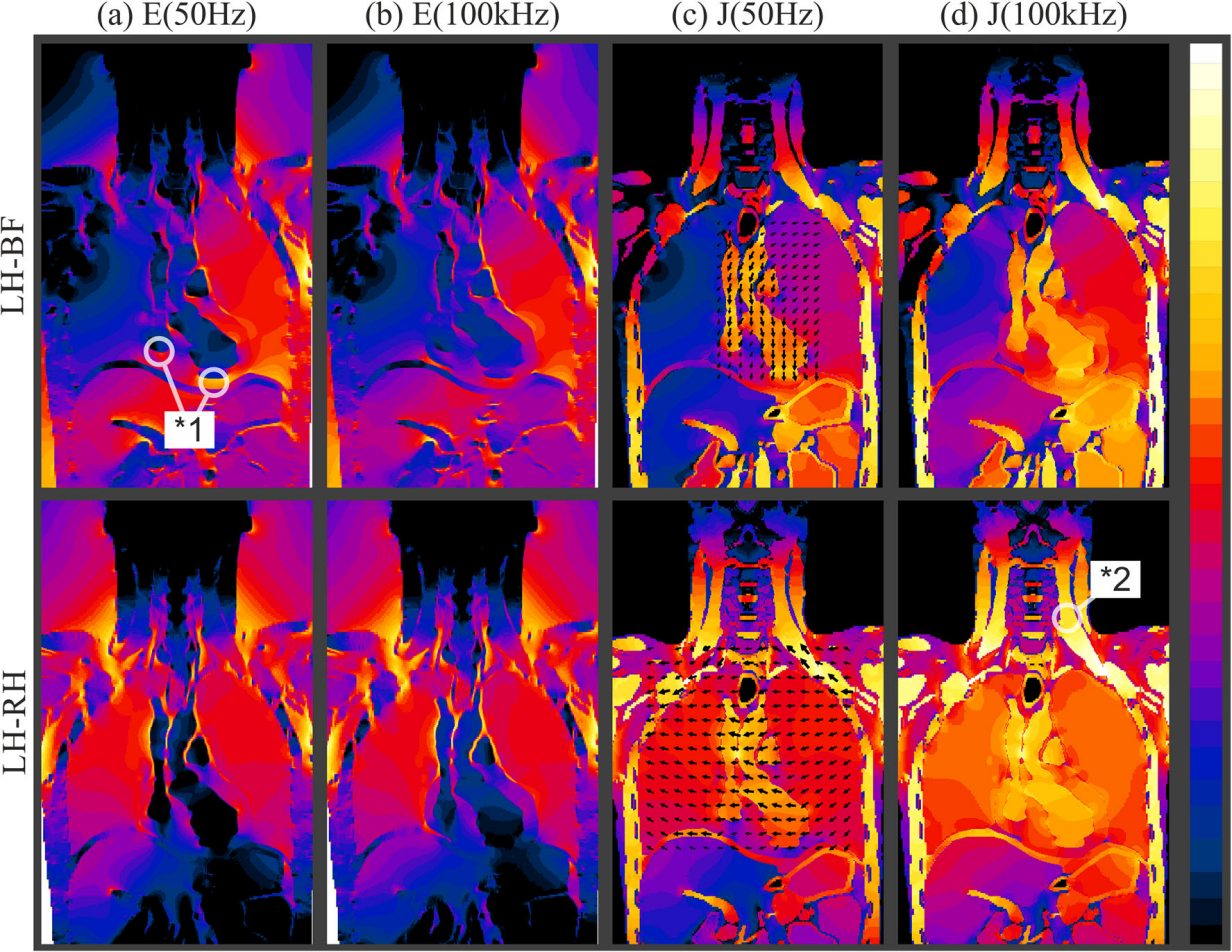
Field distributions for the current paths LH-BF and LH-RH. The amplitude of the electric field E for 50 Hz (**a**) and 100 kHz (**b**) is shown from 0 (bright) to 30 dB (dark). The current density J for 50 Hz (**c**) and 100 kHz (**d**) is shown from 0 (bright) to 40 dB (dark). The black arrows show the current density direction and amplitude. Additional markers and highlights are explained in this section’s running text

Sam [19] explained the lower HCFs for RH-xF in comparison with the LH-xF with basic human anatomy. He suggests that the asymmetric position of the ventricles lead to this effect. Figure 4 indicates a reasonable explanation as to why the numerical body model reveals similar *F*_99*Num*_ and *F*_*SamNum*_ for all longitudinal current paths. In case of longitudinal currents, the current enters the heart lumen through the blood vessels (subclavian artery/vein, aorta, and vena cava; cf. Fig. 4 LH-BF black arrows) which are in the thorax center or symmetric to the median plane. The current is then driven into the heart lumen and through the heart muscle downwards to the feet/foot/seat. It enhances the EF in the heart muscle of, e.g., the right ventricle (cf. Fig. 4 *1) and is a major determinant of *E*_99*Heart*_ and therefore *F*_99*Num*_. The described current-channeling effect is present at all longitudinal current paths (xH-xF and PS-xH) and only loses influence on *F*_99*Num*_ at 100 kHz. At these higher frequencies, the heart’s offset to the left reduces *F*_99*Num*_ of the RH and BH current paths (6% at maximum) in comparison to LH current paths and lower frequencies. This is due to the increased homogeneity of EF in the trunk at these frequencies (cf. Fig. 4, 100 kHz panels), which reduces the channeling effect.

Assuming the current-channeling effect was attenuated or not present during Sam’s [19] cadaver measurements, the anatomic asymmetry of the human heart would increase its influence on the HCF and explain the different results. There are some possible reasons why Sam did not recognize a current-channeling effect: The numerical body model used has no heart valves, which change the electrical conditions within the heart. Since the conductivity of the heart valves are lower than that of blood, closed heart valves could increase the impedance between the atria and the ventricles and thereby limit the current-channeling effect in a real human heart. Closed or incompletely open heart valves of the cadaver in Sam’s study would therefore lead to an underestimated HCF value. Likewise, the absence of the heart valve in the numerical body model could overestimate the HCF. The detection of impedance changes due to opening or closure of the heart valves is also being used in heart valve prosthesis [50].

A second explanation for the differences is connected to the conductivity of the blood. It is not, a priori, surprising that cadaver tissue properties should deviate, possibly significantly, from values of living tissue. The conductivity of the blood changes post mortem due to the coagulation process. Sam [23] performed his experiments on cadavers up to 17 h post mortem. According to Freiberger [13], the total body impedance increases rapidly in the first hours after death which might be a result of a decrease of the body temperature and tissue conductivites. Haemmerich et al. [51] considered the blood occlusion post mortem as the main reason for changes in conductivity and therefore also body impedance. A lower blood conductivity would also attenuate the current-channeling effect and could subsequently lead to an underestimation of the HCF. In the future, research simulations should be done with the body model by using post-mortem blood conductivity to prove this effect.

Additional research using body models with heart valves and comprehensive examinations of different blood conductivities could clarify to what extent the explanations hold for the differences of LH-xF vs. RH-xF longitudinal current paths. Nevertheless, the numerical model represents at least the worst-case approach for these current paths, if not the real case. It is therefore a significant addition to the current knowledge on HCFs.

### Numerical HCF of transversal current paths

Table 4 is the continuation of Table 3 and shows the *F*_99*Num*_ determined in this study for current paths (LH-RH, LF-RF, and xT-xH) which create a current flow across the trunk of the human body (transversal current).

**Table 4:** *F*_99*Num*_ according to (2) for current paths generating transversal currents

Path	*F*_*Sam*_ at 50 Hz	*F*_*SamNum*_	*F*_99*Num*_				
		50 Hz	50 Hz	1 kHz	10 kHz	100 kHz	DC
LH-RH	0.40	0.65	0.63	0.63	0.64	0.69	0.53
PT-RH	0.30	0.55	0.455	0.450	0.447	0.463	0.416
PT-LH	0.70	0.58	0.573	0.568	0.587	0.624	0.528
AT-RH	1.30	1.40	1.952	1.910	2.018	2.180	1.828
AT-LH	1.50	1.59	2.027	1.953	2.054	2.201	1.990
RF-LF	0.04	0.02	0.026	0.028	0.030	0.037	0.027

**Fig. 5:**
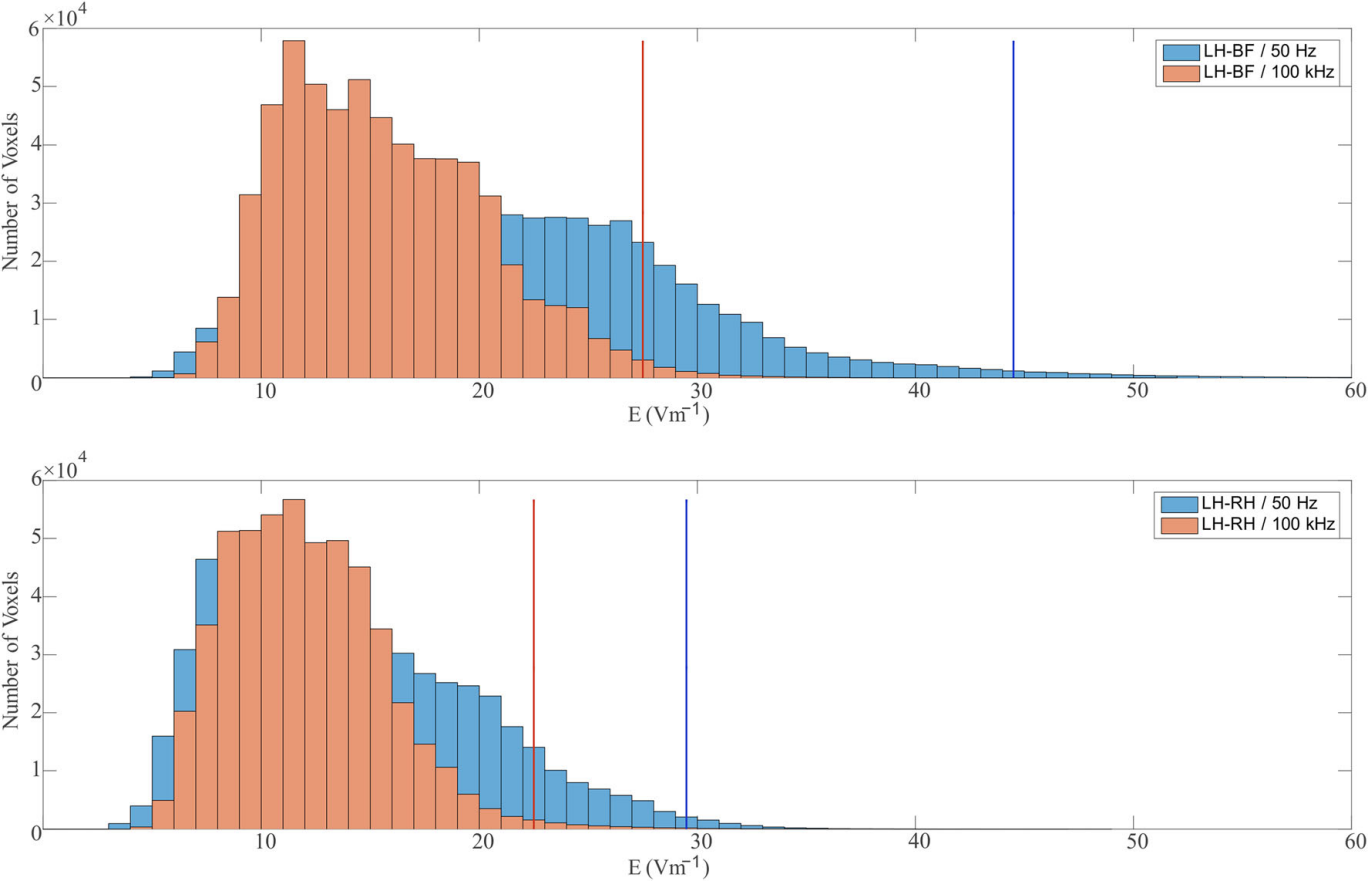
Histogram of the EF distribution in the ventricle myocardium for the current paths LH-BF and LH-RH at 50 Hz and 100 kHz. The dotted lines mark the 99th percentile (*E*_99*Heart*_) of the distribution in the corresponding color

In the path for Fig. 4, no obvious current channel is present for the LH-RH current path. The EF histograms in Fig. 5 confirm this impression. The distribution is much narrower for the LH-RH current path in comparison to LH-BF. This means a more homogenous EF distribution is created by the LH-RH current path. Consistently, the numerical imitation of Sam [19] (*F*_*SamNum*_) is in stronger accordance with Sam’s cadaver measurements (at 50 Hz) in transversal current paths than in longitudinal current paths. Meaning that, *F*_*SamNum*_ and Sam’s [19] values both show the tendency of a higher HCF for xT-LH current paths and also comply in absolute values.

*F*_99*Num*_ varies considerably among the various transversal current paths. For xT-xH current paths, *F*_99*Num*_ depends extremely on the xT contact electrode position, due to the proximity of the electrode to the heart. The dependency was found because Sam [19] did not give specific anatomical description of his electrode placement and we therefore changed the electrodes’ positions in the range of 2 cm (any direction). The electrode position shown in Fig. 1 leads to the highest *F*_99*Num*_, which are presented in Table 4. Position adjustments in the range of 1–2 cm (any direction) can influence *F*_99*Num*_ in the order of 0.5. Therefore, a safety margin should be added when using those *F*_99*Num*_, although the results are already exceeding the IEC [1] values up to 0.65 at 50 Hz. Scrutinizing the electrode position dependency of *F*_99*Num*_ could contribute to VF assessment and research needs are hereby addressed.

The strong deviation between *F*_99*Num*_ and *F*_*SamNum*_ for AT-xH occurs because of the proximity of the electrode to the heart. This creates inhomogeneity of the EF within the myocardium. As described in the methods, a possible explanation for our results differences with respect to Sam’s original measurements may stem from the averaging effect due to the probe size (160 mm). Our results, therefore, should be further investigated and given special consideration in future risk assessments of AT-xH current paths.

### HCF frequency dependency

The results for longitudinal current paths reveal nearly no influence of the frequency on the HCF (cf. Table 3). *F*_99*Num*_ deviates 7% from 1.0 at maximum, between 50 Hz and 10 kHz the deviation is even smaller (3% at maximum). This indicates that the HCF for longitudinal current path is not frequency dependent.

For transversal current paths *F*_99*Num*_ changes with the frequency, i.e., LH-RH *F*_99*Num*_ increases by 0.16 and AT-LH *F*_99*Num*_ increases by 0.21 from DC to 100 kHz. The main changes are noticeable between DC and 50 Hz and 10 kHz and 100 kHz. In contrast to the longitudinal current paths, no obvious current channels are visible in the trunk of the body model for the transversal current paths (e.g., cf. Fig. 4 LH-RH black arrows). The frequency dependency of *F*_99*Num*_ is therefore most likely due to the increasing homogeneity of the EF in the trunk. As an example of that, the EF distribution in the ventricle myocardium is shown in Fig. 5 for the current paths LH-BF and LH-RH at 50 Hz and 100 kHz. At 100 kHz, the histograms are narrower than at 50 Hz, meaning that there is less variance in the distribution of the EF strength. Further it is noticeable that *E*_99*Heart*_ decreases with the frequency for both current paths. *E*_99*Heart*_ of LH-RH decreases less with the frequency in comparison with LH-BF and therefore, according to (2), *F*_99*Num*_ increases with the frequency.

This new knowledge can be put into the context of the frequency dependency of the VF threshold, for example, given in IEC [14] and IEC [52]. The VF threshold rises roughly linearly with the frequency [53, 54]. This means that with increasing CC frequency, the myocardial cells are less sensitive. At CC frequencies above 100 kHz, myocardial tissue is therefore more likely to be burned before it is excited [8]. Note that cardiac ablation is routinely performed in humans using 500 kHz and rarely induces VF [8, 55]. Taking that into account IEC [15] introduced the so-called frequency factor, which allows higher CC amplitudes for higher frequencies. However, to date frequency-dependent HCF values have not been considered. The results of this study suggest frequency dependent HCFs for transverse current paths. For longitudinal current path, frequency correction seems not necessary.

## Conclusion

This study presents computational methods for dosimetric studies on CCs by providing an extendable and adaptable body model. The developed model features validated total body impedances, considers skeletal muscle impedance anisotropy, and includes validated skin impedance models, which take the frequency and the touch voltage into account. Both validations were conducted using in vivo data presented in IEC 60479-1 [1] which was obtained by measurements on 100 living persons at 25 V AC 50 Hz in dry conditions with large surface areas of contact. Due to strong muscular effects, higher touch voltages (up to 200 V) were determined from only one human as the mean value of 6 measurements at each touch voltage [18]. Due to the extensive validation, the numeric body model described in this study is applicable to a wide range of accident scenarios. These accident scenarios could contain any frequency between DC and 1 MHz, smaller or bigger contact areas and different contact positions.

The developed skin model has the advantage of controlling the contact area size exactly and it controls the electrical skin properties at the contact area locally, which is necessary because these properties change locally in case of CCs. When using the skin model in further studies, it should be considered that here, the skin of the hands was used for the validation. Certain body areas might have thicker or thinner skin and therefore other electrical properties. The skin model was validated for contact dimensions of 10 cm. The derived conductivity and relative permittivity values, however, allow impedance estimations for smaller or bigger contact areas. In case of uncertainty concerning the skin properties, impedance measurements would be necessary to calculate an adapted skin model using the methods presented above.

The presented HCF assessment is one of the major applications for the body model. It helps to understand the risk dependence of VF on the CC path and frequency. The results reveal HCFs from DC to 100 kHz and show frequency-dependent HCFs for transversal current paths, which are not considered in current standards like IEC [15] so far. Additionally, the model enables HCF calculations for special applications where individual current paths are in question. The comparison of Sam’s cadaver study and the numerical approach revealed that the blood conductivity, the heart valves, and the myocardial anisotropy need to be further researched in order to evaluate their exact influence on the HCFs for longitudinal current paths. Until such, research is performed and published, the results presented by Sam and published by IEC 60479-1 shall continue being the guiding information for cardiac risk and for estimating heart factor values.

A second major application field for the developed model is the assessment of certain tissues or particular organs. In Fig. 4, the marker *2 shows the current density in the carotid arteries and jugular veins as being highly significant. Thus, brain current factors should be of interest in upcoming studies to explain injuries affecting the brain following electrical injuries [6]. The body models’ output datasets (electric field and current density) enable ICNIRP [20] and IEEE [21, 22] assessments for every current path and frequency from DC to 1 MHz.
